# Influence of Core Oligosaccharide of Lipopolysaccharide to Outer Membrane Behavior of *Escherichia coli*

**DOI:** 10.3390/md13063325

**Published:** 2015-05-27

**Authors:** Zhou Wang, Jianli Wang, Ge Ren, Ye Li, Xiaoyuan Wang

**Affiliations:** 1 State Key Laboratory of Food Science and Technology, Jiangnan University, 1800 Lihu Road, Wuxi 214122, China; E-Mails: wangzhou0920@126.com (Z.W.); liye@jiangnan.edu.cn (Y.L.); 2 Key Laboratory of Industrial Biotechnology of Ministry of Education, School of Biotechnology, Jiangnan University, 1800 Lihu Road, Wuxi 214122, China; E-Mails: 692507010@qq.com (J.W.); chinarenge@foxmail.com (G.R.); 3 Synergetic Innovation Center of Food Safety and Nutrition, Jiangnan University, Wuxi 214122, China

**Keywords:** lipopolysaccharide, core oligosaccharide, cell surface hydrophobicity, outer membrane permeability, biofilm formation, auto-aggregation, *Escherichia coli*

## Abstract

Lipopolysaccharides, major molecules in the outer membrane of Gram-negative bacteria, play important roles on membrane integrity of the cell. However, how the core oligosaccharide of lipopolysaccharide affect the membrane behavior is not well understood. In this study, the relationship between the core oligosaccharide of lipopolysaccharide and the membrane behavior was investigated using a series of *Escherichia coli* mutants defective in genes to affect the biosynthesis of core oligosaccharide of lipopolysaccharide. Cell surface hydrophobicity, outer membrane permeability, biofilm formation and auto-aggregation of these mutant cells were compared. Compared to the wild type W3110, cell surface hydrophobicities of mutant Δ*waaC*, Δ*waaF*, Δ*waaG*, Δ*waaO*, Δ*waaP*, Δ*waaY* and Δ*waaB* were enhanced, outer membrane permeabilities of Δ*waaC*, Δ*waaF*, Δ*waaG* and Δ*waaP* were significantly increased, abilities of biofilm formation by Δ*waaC*, Δ*waaF*, Δ*waaG*, Δ*waaO*, Δ*waaR*, Δ*waaP*, Δ*waaQ* and Δ*waaY* decreased, and auto-aggregation abilities of Δ*waaC*, Δ*waaF*, Δ*waaG*, Δ*waaO*, Δ*waaR*, Δ*waaU*, Δ*waaP* and Δ*waaY* were strongly enhanced. These results give new insight into the influence of core oligosaccharide of lipopolysaccharide on bacterial cell membrane behavior.

## 1. Introduction

**Figure 1:**
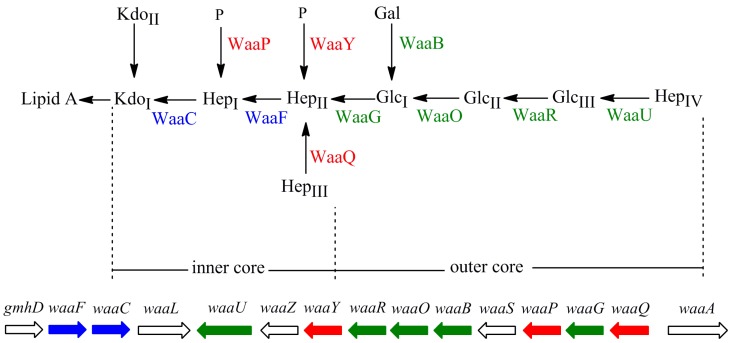
Structure and biosynthesis of the core oligosaccharide of LPS in *E. coli* W3110*.* Organization of the *waa* locus is also shown. Glycosyltransferases that construct the inner core backbone and the genes encoding these enzymes are shown in blue; enzymes that modify the structure of inner core and the genes encoding them are shown in red. Glycosyltransferases that construct the outer core and the genes encoding them are shown in green.

In most Gram-negative bacteria, lipopolysaccharide (LPS), the major component in the outer leaflet of outer membrane, provides the structural integrity of the outer membrane [1,2]. In *Escherichia coli*, LPS typically consists of a hydrophobic domain known as lipid A, a nonrepeating core oligosaccharide (core OS), and a distal polysaccharide known as *O*-antigen repeats [2]. *E. coli* strain W3110 only synthesizes LPS without *O*-antigen repeats due to the insertion of IS5 element in the *rfb* gene cluster, which encodes enzymes for biosynthesis of *O*-antigen [3]. The core OS is divided into two regions: inner core (lipid A proximal) and outer core (Figure 1). The inner core region, typically containing residues of 3-deoxy-d-manno-octulosonic acid (Kdo) and l-glycero-d-manno-heptose (Hep), is often decorated with phosphate (P). The structure of the inner core tends to be well conserved within a genus or family, suggesting its importance in outer membrane integrity. The outer core region typically contains glucose (Glc) and Hep. 

The core OS is assembled on lipid A via sequential glycosyl transfer from nucleotide sugar precursors. In *E. coli*, the chromosomal *waa* locus encodes enzymes required for biosynthesis of the core OS. The *waa* locus consists of three operons: *gmhD*, *waaQ*, and *waaA* [4,5,6,7]. The *gmhD* operon contains genes *waaC* and *waaF* required for addition of Hep. The *waaQ* operon contains eight genes necessary for biosynthesis of the outer core and its modification. The *waaA* operon only contains the structural gene *waaA* encoding the bi-functional Kdo transferase. The heptosyltransferases, WaaC and WaaF, add Hep residues in the inner core of LPS. LPS kinase WaaP and phosphatase WaaY add phosphate group to the first and second Hep residues, respectively. Heptosyltransferase WaaQ adds the third Hep to the second Hep residue. These modifications are proceeded in the strict order of WaaP, WaaQ and WaaY. Then, glucosyltransferase WaaG adds the first Glc residue, the first sugar in the outer core of LPS, to the second Hep residue; the first Glc is modified by WaaB. Next, glucosyltransferases WaaO and WaaR add the second and thirrd Glc, respectively. Finally, heptosyltransferase WaaU adds the fourth Hep to the third Glc.

The outer membrane of Gram-negative bacteria is a barrier to many antibiotics and host defense factors [8], it is also important for nutrient absorption and waste discharge of the cell. LPS, as the major molecule in outer membrane, plays important roles on membrane behavior. Mutations in LPS can alter outer membrane stability, giving rise to pleiotropic phenotype, such as changes of membrane protein expression, outer membrane permeability and cell motility [9,10,11]. Core OS of LPS is the important link between lipid A and *O*-antigen, but the detailed influence of core OS to membrane stability, permeability, biofilm formation and auto-aggregation has not been elucidated. To understand the effect of core OS on membrane behavior, a whole set of deletion mutations of single genes in the *waa* locus of *E. coli* were constructed, and their membrane behavior were investigated.

## 2. Results and Discussion

### 2.1. Construction of 10 E. coli LPS Core OS Mutant Strains and Comparison of Their LPS Structure and Cell Growth

**Figure 2:**
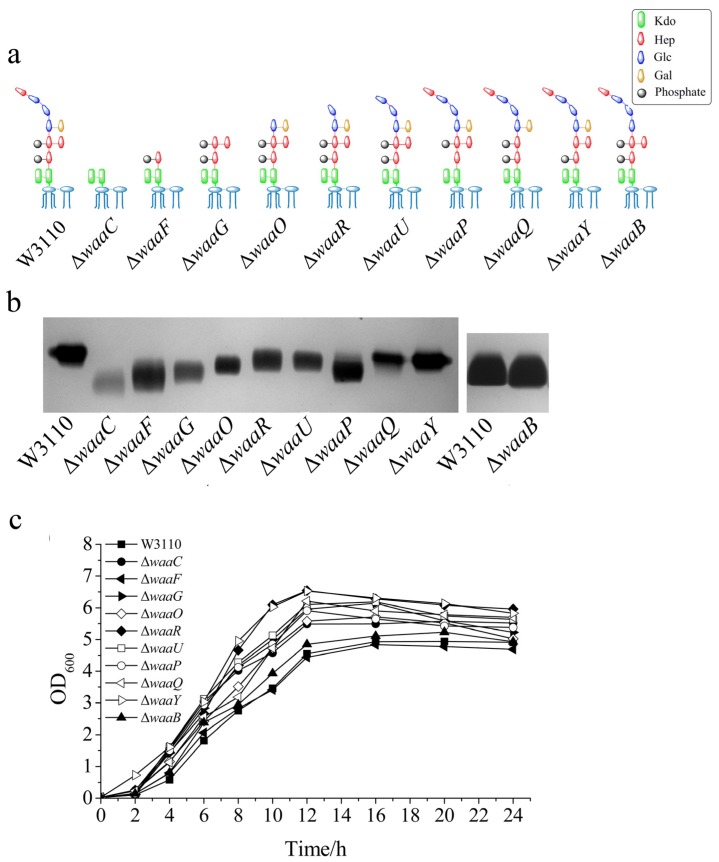
Comparison of LPS structure and cell growth of different *E. coli* strains. (**a**) The expected LPS structure; (**b**) LPS mobility on a silver stained tricine-PAGE; and (**c**) growth curve of *E. coli* W3110, Δ*waaC*, Δ*waaF*, Δ*waaG*, Δ*waaO*, Δ*waaR*, Δ*waaU*, Δ*waaP*, Δ*waaQ*, Δ*waaY* and Δ*waaB*. The same residues in different LPS structures were shown in the same color and shape. Kdo, 3-deoxy-d-manno-octulosonic acid; Hep, l-glycero-d- manno-heptose; P, phosphate; Glc, d-glucose; and Gal, d-galactose.

Ten genes involved in LPS outer core biosynthesis were individually deleted from the chromosome of *E. coli* W3110, resulting in *E. coli* mutant strains Δ*waaC*, Δ*waaF*, Δ*waaG*, Δ*waaO*, Δ*waaR*, Δ*waaU*, Δ*waaP*, Δ*waaQ*, Δ*waaY* and Δ*waaB*, respectively. The expecting structures of LPS in these 10 *E. coli* mutant strains are shown in Figure 2a. 

LPS was isolated from the above 10 *E. coli* mutant cells and analyzed by silver-stained tricine-PAGE, using LPS isolated from the wild type *E. coli* W3110 as the control (Figure 2b). LPS from all the mutant strains migrated faster than LPS from W3110, suggesting that the LPS structure was changed in these mutant cells. The migration rates of different LPS samples are related to the number and structures of the groups in the core OS (Figure 2b). Since mutants Δ*waaC*, Δ*waaF* and Δ*waaG* synthesize LPS without the outer core (Figure 2a), their LPS ran much faster than other LPS on the SDS-PAGE (Figure 2b). LPS from Δ*waaC* has the simplest structure among the 10 LPS samples, therefore, it migrated the fastest (Figure 2b). LPS isolated from Δ*waaO*, Δ*waaR*, Δ*waaU*, Δ*waaQ*, Δ*waaY* and Δ*waaB* ran only a little faster than LPS from W3110, which is consistent with that their structure contains the whole inner core and only loss a few groups in the outer core. Mutant Δ*waaP* also synthesize LPS containing the whole inner core but its LPS ran faster, possibly because the loss of phosphate group changed its negative charge or other groups cannot add in the outer core without the presence of the phosphate group [4,11]. 

To investigate the influence of the core OS on the cell growth, the 10 *E. coli* mutant strains were grown at 37 °C for 24 h, using W3110 as the control. As shown in Figure 2c, most mutant strains grew better than the control. Among the 10 mutant strains, Δ*waaF* grew the slowest, Δ*waaY* and Δ*waaR* grew the fastest. It seems that the cell growth does not depend on the length of their core OS, but does depend on the residues on the core OS. For example, Δ*waaB* grew much slower than Δ*waaY* even though their LPS are the same length. This also suggests that the galactose group in the outer core of LPS is important for cell growth.

### 2.2. The Effect of LPS Core OS on Cell Surface Hydrophobicity and Outer Membrane Permeability

**Figure 3:**
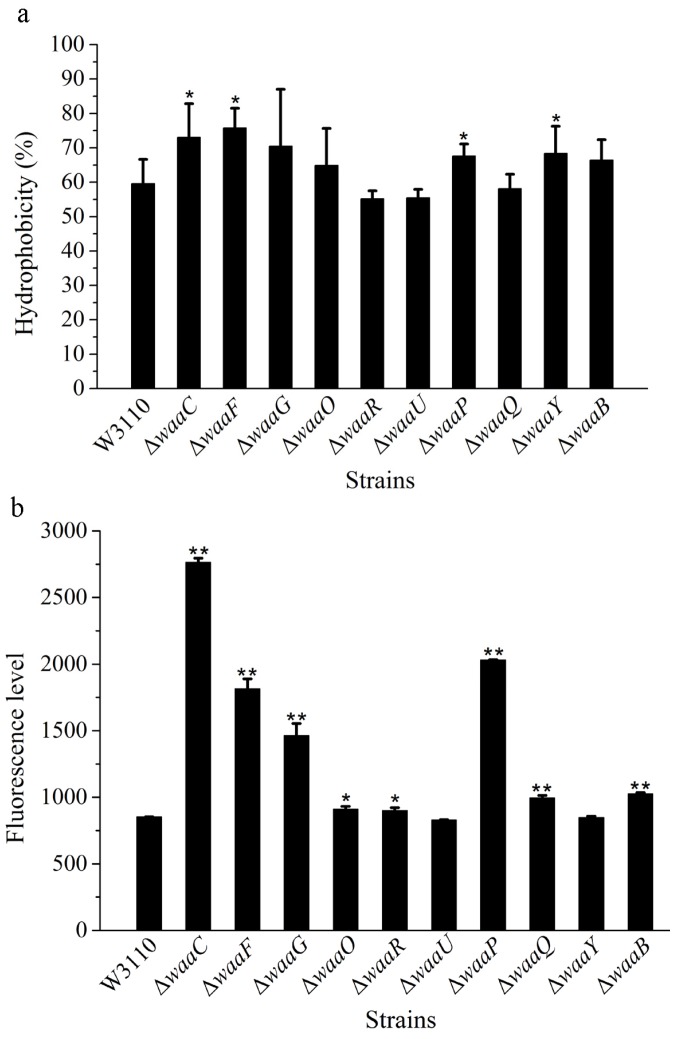
Cell surface hydrophobicity (**a**) and outer membrane permeability (**b**) of LPS core OS mutants derived from W3110. The values represent the mean ± SD of results from three independent experiments. Statistical analysis was performed using ANOVA. ** *P* ＜ 0.01 and * *P* ＜ 0.05, against the control strain W3110.

Since LPS are major components in the outer layer of the outer membrane of the cell, the structure change of the core OS might affect the cell membrane. The surface hydrophobicity and outer membrane permeability of the 10 LPS mutant strains were analyzed, using *E. coli* W3110 as the control (Figure 3). 

As an amphipathic molecule, LPS has a hydrophobic portion embedded in the outer membrane and a hydrophilic polysaccharide projected away from the cell surface [12]. The variation of sugar residues in LPS core OS can alter the distribution between the hydrophilic and hydrophobic portions of the molecule, resulting in a change in the surface hydrophobicity of the whole cell. Based on the xylene assay, the hydrophobicity of mutant cells Δ*waaC*, Δ*waaF* and Δ*waaG* was significantly increased; suggesting LPS lacking the outer core can increase the hydrophobicity of cells (Figure 3a). The hydrophobicity of mutant cells Δ*waaP* and Δ*waaY* was also significantly increased; indicating the loss of phosphate group in LPS makes the molecule more hydrophobic. 

The membrane permeability of the 10 LPS mutant strains were also analyzed, using W3110 as the control (Figure 3b). Compared with W3110, membrane permeability of Δ*waaC* increased four-fold, and membrane permeability of Δ*waaF* and Δ*waaG* was also significantly increased; suggesting that the outer core of LPS is important for the outer membrane permeability. The membrane permeability of Δ*waaP* increased significantly, but not Δ*waaY,* suggesting that the combined modification of phosphate and phosphate ethanolamine in the inner core of LPS is important for the stability of outer membranes. Several papers have reported the modulation of lipid A portion of LPS increased the outer membrane permeability of Gram-negative bacteria [13]. This study indicates that the core OS of LPS is also important for the outer membrane permeability.

The susceptibility of bacterial cells to antibiotics is closely related to LPS structure. For example, *Pseudomonas aeruginosa* cells containing smooth LPS were more susceptible to aminoglycosides than those containing rough LPS [14,15,16]; and the polysaccharide of LPS was considered to play the role in the cell permeability and ionic binding of this agents. However, in other reports, LPS core defects decreased cell’s resistance to antibiotics [17,18,19]. In this study, we analyzed the susceptibility of the 10 mutant strains to two antibiotics, erythromycin and novobiocin (Table 1). The overall patterns of susceptibility to both antibiotics were similar. Mutant cells Δ*waaC*, Δ*waaF* and Δ*waaG* was significantly sensitive to both erythromycin and novobiocin, suggesting that LPS lacking the outer core significantly changed the outer membrane structure and made the cell more fragile. Δ*waaP* also showed significant increase in susceptibility to both antibiotics, suggesting the phosphate group in the inner core is important for membrane protection. Defect of LPS increased the outer membrane permeability and hydrophobicity, which might facilitate the membrane crossing of antibiotics. The results of membrane permeability and antibiotic susceptibility are consistent, suggesting the permeability change of LPS mutants played major roles on the cell susceptibility to erythromycin and novobiocin. 

**Table 1:** Cell susceptibility analysis of *E. coli* wild type W3110, LPS mutant strains Δ*waaC*, Δ*waaF*, Δ*waaG*, Δ*waaO*, Δ*waaR*, Δ*waaU*, Δ*waaP*, Δ*waaQ*, Δ*waaY* and Δ*waaB*.

Strain	MIC (μg/mL)	
	Erythromycin	Novobiocin
W3110	>500	>500
Δ*waaC*	62.5	15.6
Δ*waaF*	62.5	31.3
Δ*waaG*	125	250
Δ*waaO*	500	>500
Δ*waaR*	>500	>500
Δ*waaU*	>500	>500
Δ*waaP*	125	15.6
Δ*waaQ*	500	>500
Δ*waaY*	>500	250
Δ*waaB*	500	>500

### 2.3. Comparison of Biofilm Formation and Auto-Aggregation of 10 E. coli LPS Mutant Strains

**Figure 4:**
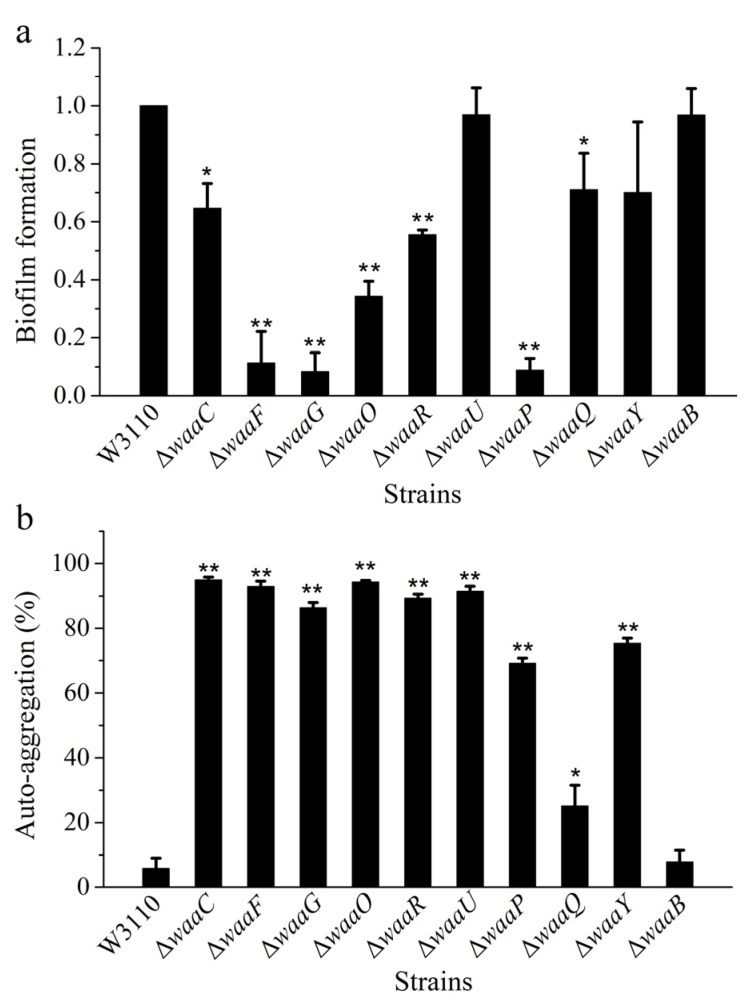
Comparison of biofilm formation (**a**) and auto-aggregation (**b**) of *E. coli* strains W3110, Δ*waaC*, Δ*waaF*, Δ*waaG*, Δ*waaO*, Δ*waaR*, Δ*waaU*, Δ*waaP*, Δ*waaQ*, Δ*waaY* and Δ*waaB*.

LPS locates on the bacterial cell surface, and it is closely related to biofilm formation. However, the exact role of the core OS on biofilm formation is not well understood. In order to compare biofilm formation ability of different LPS mutant strains, *E. coli* strains W3110, Δ*waaC*, Δ*waaF*, Δ*waaG*, Δ*waaO*, Δ*waaR*, Δ*waaU*, Δ*waaP*, Δ*waaQ*, Δ*waaY* and Δ*waaB* were grown in a 96-well microtiter plate for 24 h, and the formed biofilm was stained with crystal violet and measured by the spectrophotometer (Figure 4a). Mutant cells Δ*waaU* and Δ*waaB* have the ability for biofilm formation similar to the control W3110, mutant strains Δ*waaF*, Δ*waaG*, and Δ*waaP* almost lost their ability for biofilm formation, and the ability for biofilm formation of mutant cells Δ*waaC*, Δ*waaO*, Δ*waaR*, Δ*waaQ*, and Δ*waaY* were also significantly decreased. The big difference of biofilm formation between Δ*waaC* and Δ*waaF* suggests that the length of LPS core OS is not the only factor that affect the biofilm formation. There was no obvious positive relevance between the hydrophobicity and biofilm formation of *E. coli* core OS mutations [20]. Biofilm formation was a complex process, it was affected by the cell membrane, and secretion of extracellular polymeric substances; the adhesion ability of cell was the important factor and force in the process of biofilm formation. In some bacteria, LPS provides the initial adhesion for the appropriate coverage of substratum and the construction of biofilm matrix [21]. For example, LPS is related to biofilm formation of *Azospirillum brasilense* or *P. aeruginosa* on hydrophilic and hydrophobic surfaces [22,23,24]. LPS is involved in initial adhesion on surface for biofilm formation, and defect in LPS might lower biofilm formation.

Another factor that affect the adhesion ability of bacteria is auto-aggregation. Relationships between adhesion ability and surface hydrophobicity [23,25] or auto-aggregation [26,27] have been reported. In this study, auto-aggregation ability of *E. coli* strains W3110, Δ*waaC*, Δ*waaF*, Δ*waaG*, Δ*waaO*, Δ*waaR*, Δ*waaU*, Δ*waaP*, Δ*waaQ*, Δ*waaY* and Δ*waaB* were investigated (Figure 4b). Compared with the control W3110, all LPS mutant strains except for Δ*waaQ* and Δ*waaB* showed strong ability of auto-aggregation. Auto-aggregation is mediated by the autotransporter Ag43 located in cell membrane [28], the short LPS synthesized in core OS mutants might exposed Ag43, leading to the increase of auto-aggregation. At the same time, defect in LPS structure might allow demasking of surface adhesins, promoting bacterial aggregation.

## 3. Experimental Section

### 3.1. Bacterial Strains and Growth Condition

*E. coli* K-12 W3110 was used for mutant construction and as the background strain for all experiments, and the mutants constructed by Red recombination system [28,29,30]. Bacteria were grown in LB medium or on LB agar at 37 °C or 30 °C. When necessary, the medium was supplemented with ampicillin (100 μg/mL), kanamycin (30 μg/mL) or chloramphenicol (30 mg/mL) for plasmid maintenance or strain selection. The wild type *E. coli* W3110 and all core OS mutants were grown in LB medium at 37 °C with agitation at 200 rpm. Each culture was inoculated with overnight culture and adjusted the OD_600_ to the range of 0.02–0.05. Cultures were incubated at 37 °C with agitation at 200 rpm, and OD_600_ of samples taken every 2 h was measured.

### 3.2. DNA Manipulation

The genes of core OS were independently replaced by a non-polar kanamycin resistance cassette (the *kan* gene from pKD13). For example, to knockout the *waaC* gene, the upstream homologous fragments were amplified by the primer pair *waaC*-U-F and *waaC*-U-R, and the downstream homologous fragments were amplified by the primer pair *waaC*-D-F and *waaC*-D-R. Then, the two fragments were linked with the *kan* gene from plasmid pKD13 into plasmid pBluescript II SK(+). The DNA segment was then PCR amplified using the primer pair *waaC*-U-F and *waaC*-D-R, and transferred into the recipient cell by electroporation. The first recombination mutant would be obtained with the help of Red recombination plasmid, pKD46. Then the removal of *kan* gene was done using plasmid pCP20 containing Flp, after the removal of pKD46 at 42 °C. The successful insertion and deletion of the resistance cassette was confirmed by PCR analysis and loss of resistance. The *waaC* mutant without any resistance was obtained after the removal of pCP20 at 42 °C. Other genes were deleted in the same way, using different pairs of primers (Table 2). Mutations were verified by PCR amplification of the mutated gene from the chromosome. An overview of the plasmids and mutants used in this study is shown in Table 3.

**Table 2:** Primers for PCR (Polymerase Chain Reaction) amplification used in this study. The restriction enzyme sites were underlined.

Primers	Sequence (5′→3′)	Restriction Enzyme
*waaC*-U-F	CCGCTCGAGTAAATCAAGCAAGCCTAT	*Xho*I
*waaC*-U-R	CGGAATTCCAGTCAAGCAGTTTTGGA	*Eco*RI
*waaC*-D-F	CCCAAGCTTATCCGTCAGGCTTCCTCT	*Hin*dIII
*waaC*-D-R	AAAACTGCAGCTGGTTGCCCTGTATGGT	*Pst*I
*waaF*-U-F	CCGCTCGAGAGAACCAGGCTTTAC	*Xho*I
*waaF*-U-R	CGGAATTCAGAGGAAGCCTGACGGAT	*Eco*RI
*waaF*-D-F	CCCAAGCTTAGCTCTTATGCGTCGCGATTCAG	*Hin*dIII
*waaF*-D-R	AAAACTGCAGTGCTACGCTGGCTTATC	*Pst*I
*waaG*-U-F	CCGCTCGAGGCGAGGCTATCAGGTTGT	*Xho*I
*waaG*-U-R	CGGAATTCTATGGGACTTAACTGGCACCTG	*Eco*RI
*waaG*-D-F	CCCAAGCTTCTCGGCGTGCGGAGCAATGT	*Hin*dIII
*waaG*-D-R	AAAACTGCAGCACTCAGGCGATGAATAG	*Pst*I
*waaO*-U-F	CCGCTCGAGTATCAGTGCCGATTGTGTC	*Xho*I
*waaO*-U-R	CGGAATTCTCCTGGAAAAACACCTGCT	*Eco*RI
*waaO*-D-F	CCCAAGCTTAGGATTTAGCAACTATC	*Hin*dIII
*waaO*-D-R	AAAACTGCAGTGGCAGGAAATGAGTCC	*Pst*I
*waaR*-U-F	CCGCTCGAGAGGGTAGCATTGTGGACT	*Xho*I
*waaR*-U-R	CGGAATTCTGTGATGGAAACACCTAC	*Eco*RI
*waaR*-D-F	CCCAAGCTTCAACTAAACCGTGGCACAA	*Hin*dIII
*waaR*-D-R	AAAACTGCAGCTCCTGCTATAATTCCTG	*Pst*I
*waaU*-U-F	CCGCTCGAGCTTCAAGACATCAGTGCAG	*Xho*I
*waaU*-U-R	CGGAATTCGTGAAAAGTTCCTAAGC	*Eco*RI
*waaU*-D-F	CCCAAGCTTTCCCTCGCATTTAATTTGGTCC	*Hin*dIII
*waaU*-D-R	AAAACTGCAGGGGGATTGGACTCAGTGATGTG	*Pst*I
*waaP*-U-F	CCGCTCGAGGGTGGTTTAGATGGTTG	*Xho*I
*waaP*-U-R	CGGAATTCACTCAGGCGATGAATAG	*Eco*RI
*waaP*-D-F	CCCAAGCTTTTTTGGGATGCCTTTA	*Hin*dIII
*waaP*-D-R	AAAACTGCAGAATCCTTTGCGTTGTGTT	*Pst*I
*waaQ*-U-F	CCGCTCGAGAGCACGTCAAAGTAAGT	*Xho*I
*waaQ*-U-R	CGGAATTCCTTTATGACCAGGATTT	*Eco*RI
*waaQ*-D-F	CCCAAGCTTAATTATGATCGTGGCG	*Hin*dIII
*waaQ*-D-R	AAAACTGCAGTGGTTGGTATGGGACTT	*Pst*I
*waaY*-U-F	CCGCTCGAGCAGGAATTATAGCAGGA	*Xho*I
*waaY*-U-R	CGGAATTCCGGTAAAAACAACCAAGTC	*Eco*RI
*waaY*-D-F	CCCAAGCTTCCCATCGTGGTAACTTCA	*Hin*dIII
*waaY*-D-R	AAAACTGCAGCTTTCAAACGCCGCAT	*Pst*I
*waaB*-U-F	CCGCTCGAGATAGCGTTTATCGGCGAAGC	*Xho*I
*waaB*-U-R	CGGAATTCGCCATCCCTTATCCATTTTG	*Eco*RI
*waaB*-D-F	CCCAAGCTTTTTCCCAATGACCCTACT	*Hin*dIII
*waaB*-D-R	AAAACTGCAGATCTCCCGGTTGATACAGA	*Pst*I
*Fkan*-F	CGGAATTCGTGTAGGCTGGAGCTGCTTCG	*Eco*RI
*Fkan*-R	CCCAAGCTTGCCATTAATTCACTGATCAG	*Hin*dIII

**Table 3:** Bacterial strains and plasmids used in this study.

Strains or Plasmids	Description	Source
Strains		
W3110	Wild-type *E. coli*, F−, λ−	Laboratory strain
W3110/pKD46	W3110 transformed by pKD46	This work
Δ*waaC*	W3110 Δ*waaC*	This work
Δ*waaF*	W3110 Δ*waaF*	This work
Δ*waaG*	W3110 Δ*waaG*	This work
Δ*waaO*	W3110 Δ*waaO*	This work
Δ*waaR*	W3110 Δ*waaR*	This work
Δ*waaU*	W3110 Δ*waaU*	This work
Δ*waaP*	W3110 Δ*waaP*	This work
Δ*waaQ*	W3110 Δ*waaQ*	This work
Δ*waaY*	W3110 Δ*waaY*	This work
Δ*waaB*	W3110 Δ*waaB*	This work
Plasmids		
pKD46	ParaBγβ exo, Repts,AmpR	[30]
pKD13	oriR6K, FRT Kan^R^ FRT, Amp^R^	[30]
pCP20	FLP+, λ cI857+, λpRRepts, CamR, AmpR	[30]
pBlueScript II SK+	Cloning vector, ColE1, *lacZ*, AmpR	Stratagene

### 3.3. LPS Preparation and Analysis

LPS was isolated by a modification of the phenol-chloroform method [31]. Wet cells of 1.5 mL overnight culture was gathered by centrifugation at 12,000 rpm for 3 min, the cell pellet were suspended in 100 μL TAE buffer and mixed with 200 μL of the solution I (3% SDS, 0.6% Tris and 6.4% of 2 M NaOH). The mixture was heated at 100 °C for 15 min and then mixed with phenol-chloroform (1:1, v/v), the supernatant was transferred to a new centrifuge tube after centrifugation at 12,000 rpm for 3 min, and duplicated the extraction step by phenol-chloroform three times. Then, the supernatant was transferred to a new tube and mixed with 200 μL H_2_O and 50 μL sodium acetate (3 M, pH 5.2). LPS was precipitated by adding two volumes of ethanol. The precipitate was dissolved in the 200 μL of solution II (50 mM Tris-hydrochloride, pH8.0 and 100 mM sodium acetate), and precipitated with two volumes of ethanol. The final LPS precipitate was dissolved in 50 μL H_2_O. The LPS sample was applied to SDS-PAGE gel, and stained with silver.

### 3.4. Cell Surface Hydrophobicity Assay

Cell surface hydrophobicity was measured according to the published method [32]. Briefly, after growing in LB overnight, bacterial cells were harvested by centrifugation at 12,000 rpm for 5 min, washed twice with potassium phosphate buffer (50 mM, pH 7.2), and resuspended in the same buffer to OD_600_ about 0.5. The cell suspension (5 mL) was mixed with 1 mL of xylene, and then the mixture was vigorously vibrated for 120 s and incubated for 1 h at room temperature. Changes in absorbance of aqueous phase due to bacterial adhesion to xylene were measured at 660 nm. Cell surface hydrophobicity wascalculated by [(A_0_ − A_1_)/A_0_] × 100, where A_0_ and A_1_ are absorbencies of the sample before and after it is mixed with xylene.

### 3.5. Membrane Permeability Assay

The NPN access assay was used to assess outer membrane permeability [33]. Briefly, *E. coli* were grown, harvested, and suspended as described above. After washed and resuspended in PBS (20 mM, pH 7.4), the value of OD_600_ was adjusted to 0.5 with the same buffer. Then, 1.92 mL of cell suspension was mixed with 80 μL NPN (1 mM) by inversion of the cuvette immediately prior to fluorescence monitoring. Fluorescence was measured using a Fluorescence Spectrophotometer (650-60, Hitachi, Japan), with slit widths set to 5 nm and excitation and emission wavelengths set to 350 and 420 nm, respectively.

### 3.6. Antibiotic Susceptibility Assay

Antibiotic susceptibility minimum inhibitory concentrations (MIC) were determined by 2-fold serial dilutions method of novobiocin and erythromycin (from 200 to 7.6 μg/mL) using 96-well plate. Each plate was then inoculated from an overnight culture and incubated with shaking at 37 °C. Growth was scored as positive if the culture was visibly turbid. Tests were performed in triplicate and repeated on two separate occasions.

### 3.7. Quantification of Biofilm Formation

For qualitative and quantitative analysis of biofilm formation, the biofilms of *E. coli* W3110 and the core OS mutants were assayed according to the published method with minor modifications [24,34]. This assay is based on the ability of biofilm-forming bacteria to adhere to the wells of a 24-well plate, which are subsequently visualized by staining with crystal violet. 

For quantitative analysis of the biofilm formation, the strains were cultured in a glass tube; the culture and staining methods were similar as described above for the microtiter plate assays. An overnight culture of each strains and controls, was diluted 1:100 into 1.5 mL of LB and incubated stationary at 37 °C for 24 h. The tubes were then rinsed twice with phosphate buffered saline and stained with 2 mL of 0.01% crystal violet for 20 min. After being washed, tubes were air-dried and destained with 2 mL of 30% acetic acid for 15 min. The OD_560_ readings were recorded of each well about the rinsed crystal violet. Each biofilm plate assay was performed using five replicates. 

### 3.8. Auto-Aggregation Assay

Auto-aggregation assays were performed as described with slight modification [35,36]. Overnight cultures of the target bacterium in glass tube were diluted 1:100 into 5 mL LB medium and grown to the early stationary phase at 37 °C. The cultures OD_600_ were adjusted to 2.0 by dilution with 5 mL LB medium in a glass tube. The medium and cultures were mixed well by vortexing for 20 s and incubated at 37 °C. The absorbance of the upper part of culture in each tube was measured after 2 h. A decrease in absorbance at 600 nm was indicated as the auto-aggregation. Auto-aggregation ability was calculated using [(A_0_ − A_1_)/A_0_] × 100%, where A_0_ and A_1_ are the absorbance of the cultures at 0 h and after 2 h intervals.

## 4. Conclusions

LPS, the major molecules in the outer membrane of Gram-negative bacteria, play important roles on membrane characteristics of the cell. In this study, the relationship between the core OS of LPS and the membrane stability was investigated, using 10 *E. coli* LPS mutant strains. These strains have provided means to directly compare LPS in the same background, eliminating confounding factors present when using different bacteria to compare LPS structures. Compared to the wild type strain, W3110, the cell surface hydrophobicity, membrane permeability, the abilities of biofilm formation and auto-aggregation of most mutant cells changed; and these changes closely related to the position and numbers of saccharides in the core region of LPS, revealing the effect of core OS of LPS on membrane behavior of the cells. Many *E. coli* K12 strains have been developed for inexpensive and high-level expression of various products, changes on membrane permeability and auto-aggregation caused by changes on core OS of LPS might further increase the productivity. Membrane permeability and biofilm formation are closely related to survival of pathogenic bacteria; the information on membrane behavior of different LPS mutants provided in this study should be useful for developing new strategies to control and kill pathogenic bacteria [37,38,39,40,41].
